# Changes in procalcitonin and presepsin before and after immunoglobulin administration in septic patients

**DOI:** 10.1186/cc14142

**Published:** 2015-03-16

**Authors:** T Ikeda, S Ono, T Ueno, H Tanaka, S Suda

**Affiliations:** 1 Hachiouji Medical Center, Tokyo Medical University, Tokyo, Japan

## Introduction

The potentially envisaged actions of intravenous immunoglobulin (IVIg) on severe infectious disease include: virus or toxin neutralizing action; opsonic effect; complement bacteriolytic activity; and enhancement of sensitivity to antibiotics. In the case of severe infectious disease, antibiotics are often supplemented with administration of IVIg.

## Methods

The changes in sepsis markers (procalcitonin, presepsin, interleukin-6, C-reactive protein) followed by IVIg administration were investigated in severe sepsis or septic shock patients. The subjects were 410 patients admitted to an ICU with a diagnosis of severe sepsis or septic shock and from whom informed consent had been obtained for the present study. IVIg was administered intravenously for 3 days (5.0 g/ day) and measurements were undertaken before administration (day 1), on the day after completion of administration (day 4), and on day 7. The items measured were procalcitonin, presepsin, IL-6, and CRP. The effect of IVIg administration on these markers was then studied. The IVIg studied was polyethylene glycol-treated human immunoglobulin injection fluid (2.5 g, 50 ml, one vial).

## Results

The patient APACHE II score were 24.9 ± 8.2, the SOFA score 9.1 ± 3.7, and the survival rate after 28 days 83.4%. The values before IVIg administration were: procalcitonin 36.0 ± 463.3 (median 110) ng/ml, presepsin 4,548 ± 4,250 (median 3,337) pg/ml, CRP 15.6 ± 9.6 (median 14.7) mg/dl, and IL-6 13,860 ± 47,299 (median 630) pg/ml. All values were thus elevated. On the days after the completion of IVIg administration and on day 7, the level of almost all mediators (procalcitonin, presepsin, CRP, IL-6) decreased significantly. In patients with suspected severe sepsis and septic shock, presepsin reveals valuable diagnostic capacity to differentiate sepsis severity compared with procalcitonin, IL-6, CRP, and WBC. Additionally, presepsin and IL-6 reveal prognostic value with respect to 30 days and 6 months all-cause mortality throughout the first week of ICU treatment [1].

## Conclusion

The results of the present study found significant decreases of procalcitonin, presepsin and IL-6 resulting from 3 days of immunoglobulin administration, but evidence is still limited and this needs to be confirmed in larger studies.
